# Cysteine and histidine residues are involved in *Escherichia coli* Tn*21* MerE methylmercury transport

**DOI:** 10.1002/2211-5463.12341

**Published:** 2017-11-15

**Authors:** Yuka Sone, Shimpei Uraguchi, Yasukazu Takanezawa, Ryosuke Nakamura, Hidemitsu Pan‐Hou, Masako Kiyono

**Affiliations:** ^1^ Department of Public Health School of Pharmacy Kitasato University Tokyo Japan; ^2^ Faculty of Pharmaceutical Sciences Setsunan University Osaka Japan

**Keywords:** *merE*, methylmercury transport, transposon Tn*21*

## Abstract

Bacterial resistance to mercury compounds (mercurials) is mediated by proteins encoded by mercury resistance (*mer*) operons. Six *merE* variants with site‐directed mutations were constructed to investigate the roles of the cysteine and histidine residues in MerE protein during mercurial transport. By comparison of mercurial uptake by the cell with intact and/or variant MerE, we showed that the cysteine pair in the first transmembrane domain was critical for the transport of both Hg(II) and CH_3_Hg(I). Also, the histidine residue located near to the cysteine pair was critical for Hg(II) transport, whereas the histidine residue located on the periplasmic side was critical for CH_3_Hg(I) transport. Thus, enhanced mercurial uptake mediated by MerE may be a promising strategy for the design of new biomass for use in the bioremediation of mercurials in the environment.

**Abbreviations**
CCE: crude cell extract
EDTA: ethylenediaminetetraacetic acid
MF: membrane fraction
OD: optical density
PBS: phosphate‐buffered saline
TMD: transmembrane domain

Resistance to inorganic and organic mercury compounds (mercurials) is one of the most widely observed resistance phenomena in Gram‐positive and Gram‐negative bacteria. Bacterial resistance to mercurials is mediated by a number of proteins encoded by mercury resistance (*mer*) operons 1, 2, 3. Analysis of the DNA sequence of a number of *mer* operons cloned from a diverse range of bacterial species has revealed considerable similarities in genetic organization. The *mer* operons determining bacterial resistance to Hg(II) consist of a regulatory gene (*merR*), an operator/promoter (o/p) region, and at least three structural genes, namely *merT*,* merP*, and *merA*, which encode a membrane transport protein (MerT), a periplasmic Hg(II)‐binding protein (MerP), and the mercuric reductase (MerA), respectively. MerA reduces reactive inorganic Hg(II) to volatile, relatively inert Hg(0), which is under the control of the metal‐responsive positive or negative regulators MerR and MerD, respectively 1, 2, 3, 4, 5, 6. An additional gene, *merB*, encoding organomercurial lyase, is required for bacterial resistance to organomercurials 7, 8, 9.

Recently in addition to *merT* and *merC* from transposon Tn*21* and *merF* from plasmid pMER327/419, *merE* was also identified as an Hg(II) transporter gene; the putative function of the *merE* gene product is the transport of Hg(II) across the cellular membrane 10, 11, 12, 13. To date, among the four identified mercury transporters MerT, MerC, MerF, and MerE, encoded by *merT*,* merC*,* merF*, and *merE*, respectively 14, 15, 16, 17, 18, only MerE has been identified as a novel, broad mercurial transporter that governs the transport of Hg(II) and CH_3_Hg(I) 13, 19, 20. The *merE* gene at the end of the *mer* operon (*merRTPCADE*) immediately following *merD* in Tn*21* is also frequently found in many narrow‐spectrum and broad‐spectrum *mer* operons 1, 2. The predicted secondary structure of MerE has been presumed to have two transmembrane‐spanning α‐helices with a cysteine pair positioned in approximately the middle of the first helix 21. The cysteine pair is also found in the same predicted position in MerT, where it is required for Hg(II) transport 11, 22, 23. However, the mechanism of MerE‐mediated transport of mercurials, including CH_3_Hg(I), across the bacterial membrane is not yet understood in sufficient detail.

Accordingly, in the present study, we constructed six mutants with specific point mutations in the vicinal cysteine pair (Cys28 and Cys30) of *merE* to serine and in the two histidine residues (His31 and His51) of *merE* to leucine, separately or simultaneously, in order to investigate which amino acid residues are important for methylmercury transport across the cell membrane.

## Materials and methods

### Bacterial strain, plasmids, and growth conditions

*Escherichia coli* XL1‐Blue 24 bearing the pKF19k cloning vector was grown at 37 °C in Luria/Bertani (LB) medium and used for routine plasmid preparation. The medium was supplemented with 25 μg·mL^−1^ kanamycin, as necessary.

### Enzymes and reagents

The restriction enzymes, DNA ligation kit, and Taq polymerase were obtained from Takara Shuzo Corp. (Kyoto, Japan). ^14^CH_3_HgCl was obtained from Amersham (Bucks, UK). Nonradioactive mercurials were of analytical reagent grade and were purchased from Wako Pure Chemical Industries, Ltd. (Osaka, Japan).

### Plasmid construction and site‐directed mutagenesis of *merE*

**Figure 1:**
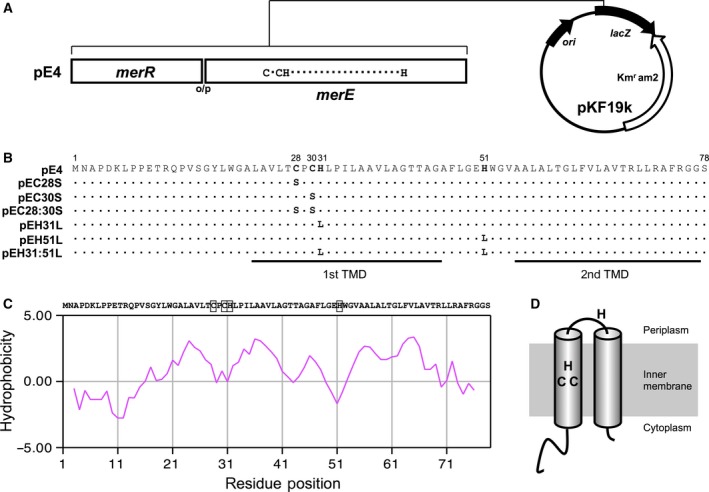
Site‐directed mutagenesis of the cysteine and histidine residues of MerE. (A) Construction of the plasmids pE4. (B) Amino acid sequences of MerE and its variants. (C) Sequence and topology predictions for MerE. (D) Topological alignments of MerE amino acids, which were separated according to their predicted hydrophobic (membrane‐spanning) elements. The putative transmembrane domains estimated using the SOSUI hydropathy program are underlined. TMD, transmembrane domain.

Plasmid pE4 13, which contained *merR*‐o/p‐*merE*, was used as the starting point for mutagenesis. The oligonucleotide‐directed dual amber‐long and accurate (ODA‐LA) polymerase chain reaction (PCR) method was used for the specific site‐directed mutagenesis of *merE* 25. Five PCR primers, that is, 5PmerE‐C28S (5′‐TGGCCGTGTTGACCAGCCCCTGCCATCTGCC‐3′), 5PmerE‐C30S (5′‐TGGCCGTGTTGACCTGCCCCAGCCATCTGCC‐3′), 5P‐merE‐C28‐30S (5′‐TGGCCGTGTTGACCAGCCCCAGCCATCTGCC‐3′), 5P‐merE‐H31L (5′‐TGCCCCTGCCTTCTGCCGATTC‐3′), and 5P‐merE‐H51L (5′‐TCCTTGGCGAGCTTTGGGGTGTTG‐3′), were used to construct the *merE* variants pEC28S, pEC30S, pEC28:30S, pEH31L, and pEH51L, respectively. The 5PmerE‐C28S, 5PmerE‐C30S, and 5P‐merE‐C28‐30S primers were used to convert the cysteine residues at positions 28 and 30 in MerE to serine, respectively. The 5P‐merE‐H31L and 5P‐merE‐H51L primers were used to convert the histidine residues at positions 31 and 51 in MerE to leucine, respectively. The 5P‐merE‐H51L primer and plasmid pEH31L were used to construct the *merE* variant pEH31:51L. Plasmids with the desired mutation were sequenced in their entirety using the dideoxy sequencing method to ensure that no other mutations had been introduced inadvertently. The recombinant *merE* plasmids were transformed into the *E. coli* strain XL1‐Blue. The structures of the relevant genes investigated in this study are shown in Fig. 1.

### Subcellular fractionation

*E. coli* XL1‐Blue cells with various plasmids, that is, pKF19k, pE4, or pE4 variants (pEC28S, pEC30S, pEC28:30S, pEH31L, pEH51L, and pEH31:51L), were grown in LB medium supplemented with 3 μm Hg(II) to an optical density (OD) of 0.8 at 600 nm. The cells were centrifuged, washed [50 mm Tris/HCl, pH 8.0, 0.15 m NaCl, 1 mm ethylenediaminetetraacetic acid (EDTA), and 10% glycerol], and suspended in 1.2 mL of the same buffer.

### SDS/PAGE and western blot analysis

Samples were added to sample buffer (62.5 mm Tris/HCl, pH 6.8, 2% SDS, 5% sucrose, and 0.005% bromophenol blue) with or without 5% 2‐mercaptoethanol‐containing dye, to a total volume of 20 μL. After boiling for 5 min, the reaction mixtures were loaded onto 12.5% SDS/polyacrylamide gels. The MerE‐His_6_‐tagged protein 13 was used as the positive control. The proteins in the gels were blotted electrophoretically onto nitrocellulose membranes using a transblot transfer cell (Bio‐Rad, Hercules, CA, USA). The membrane was immersed in 5% skim milk in PBS for 1 h to block nonspecific binding. The membrane was then incubated for 1 h at room temperature with appropriate dilutions of anti‐MerE antibodies. The procedures used to purify the MerE‐His_6_‐tagged protein and to prepare the specific antibodies have been described previously 13. The membranes were washed with PBS containing 0.1% Tween 20 and reacted with peroxidase‐conjugated anti‐rabbit IgG (Sigma‐Aldrich, St. Louis, MO, USA) for 1 h. After washing, Chemi‐Lumi One L reagent (Nacalai Tesque, Kyoto, Japan) was used to detect the antigens.

### Mercurial uptake assay

*E. coli* strain XL1‐Blue 24 cells bearing the control or recombinants described above were grown in LB broth containing 25 μg·mL^−1^ kanamycin at 37 °C overnight. The cells were harvested, suspended in the same original volume of LB broth, and grown at 37 °C until reaching an OD_600_ of 1.00. Cells at the mid‐exponential phase were harvested and resuspended in LB broth containing 100 μg·mL^−1^ chloramphenicol and 100 μm EDTA.

For the HgCl_2_ uptake assay, the cell suspension was incubated at 37 °C with 10 μm HgCl_2_. Aliquots (0.5 mL) of the suspension were harvested and washed three times using LB broth containing 100 μg·mL^−1^ chloramphenicol and 100 μm EDTA. The samples were digested with concentrated nitric acid for 1 h at 90 °C. The concentration of total mercury in the cells was measured using an atomic absorption spectrometry analyzer (Hiranuma, Ibaraki, Japan).

For the CH_3_HgCl uptake assay, the cell suspension was incubated at 37 °C with 5 μm ^14^CH_3_HgCl (2.11 GBq·mmol^−1^). Aliquots of the suspension were removed periodically and filtered through a Whatman GF/B glass microfiber filter (0.45 μm). The filters were washed three times with LB broth containing 100 μg·mL^−1^ chloramphenicol and 100 μm EDTA, and the radioactivity levels of the filters were then measured using a liquid scintillation spectrometer (PerkinElmer, Waltham, MA, USA).

### Statistical analysis

Data analysis was performed using Student's *t*‐tests performed in Microsoft Excel software.

## Results and Discussion

### Expression and cellular localization of MerE and its variants

In general, cysteine and histidine are considered as the metal‐binding amino acid residues 26. In MerT, the first cysteine pair (Cys24 and Cys25) located in the first transmembrane domain (TMD) is involved in the transport of Hg(II) through the cytoplasmic membrane 22. Tn*21‐*encoded MerE, which has recently been identified as a broad mercury transporter 13, contains two cysteine residues (Cys28 and Cys30) and two histidine residues (His31 and His51), which are thought to be the ligands for Hg(II) and/or CH_3_Hg(I). Here, six MerE variants, that is, pEC28S, pEC30S, pEC28:30S, pEH31L, pEH51L, and pEH31:51L, were constructed to investigate the specific roles of the cysteine and histidine residues in the MerE protein (Fig. 1).

**Figure 2:**
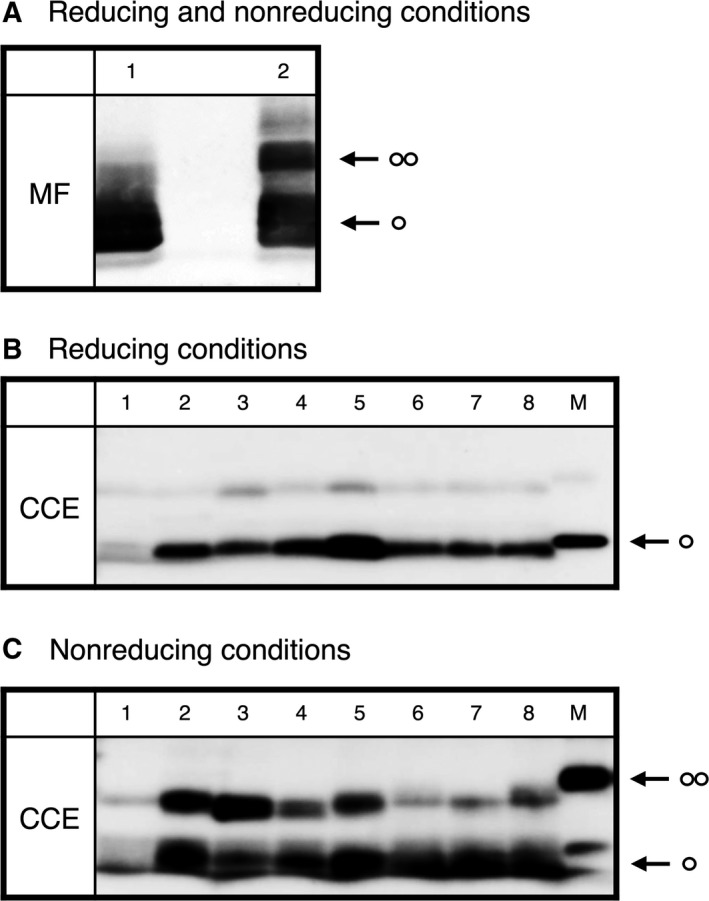
Expression of MerE and its protein variants. (A) Analysis of the expression of MerE proteins using SDS/PAGE under reducing (lane 1) and nonreducing (lane 2) conditions. (B and C) Western blot analyses of the expression of MerE variants under reducing conditions using 2‐mercaptoethanol and nonreducing conditions. Crude cell extracts (CCEs) of the transformant strain with control vector pKF19K (lane 1), recombinant plasmid pE4 (lane 2), pEC28S (lane 3), pEC30S (lane 4), pEC28:30S (lane 5), pEH31L (lane 6), pEH51L (lane 7), and pEH31:51L (lane 8) using anti‐MerE antibodies. Lane M represents the purified MerE (8 kDa). The arrows indicate the purified MerE monomer and dimer. MF, membrane fraction; CCE, crude cell extract.

The distribution profiles of MerE and its protein variants were measured in the transformants by immunoblot analysis using polyclonal anti‐MerE antibodies, followed by SDS/PAGE under reducing conditions, as previously described 13. The protein size was consistent with the size predicted based on the translation of the *merE* gene sequence. These experimental results suggest that MerE and its variant genes were successfully cloned into the bacterial cells and appropriately transcribed and translated into a protein with a molecular mass of 8 kDa (Fig. 2A, lane 1). In the presence of 2‐mercaptoethanol (under reducing condition), the MerE protein (molecular mass of 8 kDa), which reacted specifically with the anti‐MerE antibody, was identified in the crude cell extract from cells with pE4 and its variants (Fig. 2B, lanes 2–8). In the absence of 2‐mercaptoethanol (under nonreducing conditions), approximately half of the MerE protein was present as monomers, and much of MerE existed as a dimer in cells with pE4 (Fig. 2A, lane 2). These findings suggested that MerE protein may exist as a dimer on the cell membrane. The variant MerEs in cells with pEC28S, pEC30S, and pEC28:30S were present predominantly in the dimer formation (Fig. 2C, lanes 3–5). Derivatives with substitutions, that is, His31‐Leu, His51‐Leu, or His31:51‐Leu mutations, in MerE existed mainly as monomers (Fig. 2C, lanes 6–8). The mutations of the vicinal cysteines were thought to slightly affect multimer formation; however, the structure of the protein was not changed markedly (Fig. 2C).

### Role of MerE and its amino acids in the transport of mercurials

**Figure 3:**
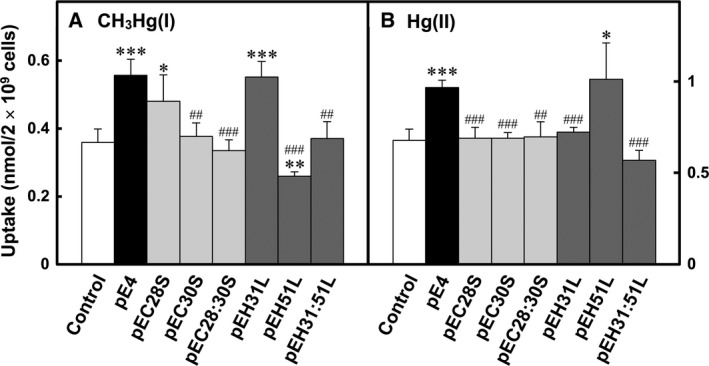
Uptake of CH_3_Hg(I) (A) and Hg(II) (B) by *E. coli* XL1‐Blue expressing MerE and its variants. *E. coli* XL1‐Blue with vector (white bar); pE4 (black bar); pEC28S, pEC30S, and pEC28:30S (light gray shading); and pEH31L, pEH51L, and pEH31:51L (gray shading) were grown, prepared, and assayed. The values are expressed as the means ± standard deviations of three measurements. *P < 0.05, **P < 0.01, ***P < 0.001 versus the control; ^#^ *P* < 0.05, ^##^ *P* < 0.01, ^###^ *P* < 0.001 versus pE4.

The uptake of CH_3_Hg(I) and Hg(II) by cells containing the pE4 plasmid and its derivatives was examined further. As shown in Fig. 3A, cells with pE4 took up significantly more CH_3_Hg(I) than control cells, which contained the cloning vector pKF19k. The cells with pE4 also took up significantly more Hg(II) than cells with the cloning vector pKF19k (Fig. 3B). Substitutions of the Cys30 residue with Ser (pEC30S), the Cys28 and Cys30 residues with Ser (pEC28:30S), and His31 and His51 residues with Leu (pEH31:51L) in MerE caused significant reductions in CH_3_Hg(I) and Hg(II) uptake compared with bacterial cells with the intact *merE* gene (pE4; Fig. 3). The His31‐to‐Leu (pEH31L) mutation in MerE reduced the uptake of Hg(II) greatly, but had no effect on CH_3_Hg(I) uptake. In contrast, the mutation of His51 to Leu (pEH51L) had no effect on Hg(II) uptake, but caused a significant reduction in CH_3_Hg(I) uptake compared with that in cells with pE4 (Fig. 3). The substitution of Cys28 in MerE with Ser (pEC28S) reduced the uptake of Hg(II) greatly and slightly reduced the uptake of CH_3_Hg(I) compared with that of cells carrying pE4. These results suggested that the cysteine pairs in the first TMD of MerE protein may have a critical role in the transport of CH_3_Hg(I) and Hg(II) across the cell membrane. For the cysteine pairs, we suggested that the Cys30 residue may be the key amino acid for mercurial transport. Moreover, the histidine residue on the periplasmic face located between the first and second TMDs of MerE was involved in CH_3_Hg(I) transport, whereas the histidine residue located next to the cysteine pairs in the first TMD of MerE was involved in Hg(II) transport.

To date, the function and structural importance of MerT 27, MerC 15, and MerF 16 have been extensively studied; however, MerE has received less attention. An outline model for the mechanism of MerT‐mediated transport of Hg(II) across the bacterial membrane has been proposed, discussed, and accepted 11, 12, 13, 14, 15, 28. Hg(II) in the periplasmic space is initially sequestered by the pair of thiol groups (Cys33 and Cys36) on MerP and subsequently transferred to the pair of thiol groups (Cys24 and Cys25) in the first TMD of MerT 12. The bound Hg(II) is then passed through the membrane to the Cys76 and Cys82 pair on the cytoplasmic face of MerT. From MerT, Hg(II) is passed to MerA, in which the substrate binding site is at the C‐terminal (Cys558 and Cys559) 28. Here, the Hg(II) is reduced to Hg(0) by MerA. Plasmids such as pDU1358 retain *merB*, encoding organomercurial lyase, in addition to *merE* and *merA*. Bacteria carrying this plasmid are thought to have the ability to take up methylmercury and cleave the organic group from the methylmercury for detoxification. Recently, Wilson *et al*. 16 reported that the mechanisms of Hg(II) transport mediated by MerT, MerC, and MerF are similar in these transporters, even though their structures in the membrane differ.

## Conclusion

To investigate the molecular function of MerE in the transport of CH_3_Hg(I) across the bacterial membrane, six *merE* variants with specific site‐directed mutations were constructed. By comparison of CH_3_Hg(I) uptake by the cell with intact and/or variant MerE, we demonstrated for the first time that the cysteine pairs (Cys28 and Cys30) within the first TMD of MerE and histidine residue (His51) on the periplasmic face located between the first and second TMDs of MerE were required for MerE‐mediated transport of CH_3_Hg(I) across the bacterial membrane, and the cysteine pairs may play a critical role in the transport of CH_3_Hg(I) and Hg(II) across the cell membrane. Thus, there was a poor correlation between multimer formation and mercurial uptake activity. We assumed that the relationship between amino acids (Cys/His) and mercurial transport activity was relevant. Currently, it is still unclear why the *merE* gene is found in Tn*21 mer* operon, which is known to confer bacterial resistance to Hg(II), but not to CH_3_Hg(I). Further studies are required to elucidate the related mechanisms.

Based on the results obtained in this study, enhanced mercurial uptake mediated by the broad‐spectrum mercury transporter MerE may be a particularly promising strategy for the design of new biomass for the bioremediation of mercurials in the environment.

## Author contributions

YS and MK designed the experiments. YS performed the experiments and wrote the manuscript. RN and YT contributed valuable suggestions to the study. SU and HPH revised the manuscript. MK conceived of and supervised the study.
